# Diffuse and Specific Tectopulvinar Terminals in the Tree Shrew: Synapses, Synapsins, and Synaptic Potentials

**DOI:** 10.1371/journal.pone.0023781

**Published:** 2011-08-15

**Authors:** Haiyang Wei, Sean P. Masterson, Heywood M. Petry, Martha E. Bickford

**Affiliations:** 1 Department of Anatomical Sciences and Neurobiology, University of Louisville, Louisville, Kentucky, United States of America; 2 Department of Psychological and Brain Sciences, University of Louisville, Louisville, Kentucky, United States of America; Tokyo Medical and Dental University, Japan

## Abstract

The pulvinar nucleus of the tree shrew receives both topographic (specific) and nontopographic (diffuse) projections from superior colliculus (SC), which form distinct synaptic arrangements. We characterized the physiological properties of these synapses and describe two distinct types of excitatory postsynaptic potentials (EPSPs) that correlate with structural properties of the specific and diffuse terminals. Synapses formed by specific terminals were found to be significantly longer than those formed by diffuse terminals. Stimulation of these two terminal types elicited two types of EPSPs that differed in their latency and threshold amplitudes. In addition, in response to repetitive stimulation (0.5–20 Hz) one type of EPSP displayed frequency-dependent depression whereas the amplitudes of the second type of EPSP were not changed by repetitive stimulation of up to 20Hz. To relate these features to vesicle release, we compared the synapsin content of terminals in the pulvinar nucleus and the dorsal lateral geniculate (dLGN) by combining immunohistochemical staining for synapsin I or II with staining for the type 1 or type 2 vesicular glutamate transporters (markers for corticothalamic and tectothalamic/retinogeniculate terminals, respectively). We found that retinogeniculate terminals do not contain either synapsin I or synapsin II, corticothalamic terminals in the dLGN and pulvinar contain synapsin I, but not synapsin II, whereas tectopulvinar terminals contain both synapsin I and synapsin II. Finally, both types of EPSPs showed a graded increase in amplitude with increasing stimulation intensity, suggesting convergence; this was confirmed using a combination of anterograde tract tracing and immunocytochemisty. We suggest that the convergent synaptic arrangements, as well as the unique synapsin content of tectopulvinar terminals, allow them to relay a dynamic range of visual signals from the SC.

## Introduction

Three main types of glutamatergic terminals have been identified in the visual dorsal thalamus of rodents, carnivores, primates, and tree shrews. All contain **r**ound synaptic vesicles, but can be distinguished based on their size as **s**mall (RS), **m**edium (RM) and **l**arge (RL) profiles. RS profiles originate from cortical layer VI [1], [2], [3], [4], [5], [6], RM profiles originate from the superior colliculus [7], [8], [9], [10] and RL profiles are contributed by primary sensory inputs, cortical layer V, or thalamocortical axon collaterals [1], [2], [7], [11], [12], [13], [14], [15], [16], [17], [18], [19].

RL profiles, such as those originating from the retina, have been described as “drivers” because the receptive field properties of postsynaptic neurons are dependent on activation of these inputs. In contrast, corticothalamic RS profiles have been described as “modulators” because their activation changes the overall responsiveness of postsynaptic neurons, but does not dramatically change receptive field properties [20]. The role of tectothalamic RM profiles is less clear, but they appear to form a third functional class of terminals [21], [22]; it has been suggested that the collective activities of multiple convergent RM inputs are critical for the formation of the receptive field properties of postsynaptic neurons. Comparisons of the excitatory postsynaptic potentials (EPSPs) elicited by stimulation of RS or RL profiles have revealed distinct forms of short-term synaptic plasticity, which are thought to reflect differences in the probability of glutamate release from each terminal type [23]. Each RL profile makes multiple synaptic contacts [7], [14], [16], [17], [24], and their stimulation elicits large amplitude EPSPs that depress when the stimulation is repeated at high frequency [25], [26], [27], [28]. In contrast, RS profiles make single synaptic contacts [16], [17], [24] and their stimulation elicits smaller amplitude EPSPs that facilitate with high frequency stimulation [23], [25], [28], [29], [30]. Recently, these features have been linked to the distribution of synapsin I and synapsin II, proteins that tether a reserve pool of synaptic vesicles [31], [32], [33], [34], [35]. These proteins are found in corticogeniculate (RS) terminals but not retinogeniculate (RL) terminals, and in synapsin I/II knockout mice the short-term plasticity of corticogeniculate EPSPs is altered, while retinogeniculate EPSPs are unchanged [36].

Unlike RS and RL profiles, the amplitudes of EPSPs elicited by stimulation of tectothalamic terminals (RM profiles) remain relatively stable at stimulation frequencies of 1–10 Hz [21], [22]. To investigate potential mechanisms responsible for the stability of RM EPSPs, we correlated morphological features of tectopulvinar terminals with the postsynaptic responses elicited by their stimulation in tissue obtained from tree shrews (*Tupaia belangeri*). In this species, tectopulvinar terminals are classified as RM profiles, but they exhibit two different types of synaptic arrangements, referred to as “diffuse” and “specific” [9], [37]. The specific projections are topographically arranged and distributed throughout both the dorsal (Pd) and central (Pc) pulvinar nucleus. The diffuse projections are nontopographic projections that only innervate the Pd. Our recent tract tracing studies indicate that the Pd and Pc project to the temporal cortex and striatum, and the Pd additionally projects to the amygdala [6], [38]. Thus, we have suggested that the specific projections relay topographic visual information from the SC to the cortex and striatum to aid in guiding precise movements, while the diffuse projection relays nontopographic visual information from the SC to the amygdala to alert the animal to potentially dangerous visual images.

In the current study, we characterized the length of synaptic contacts made by diffuse and specific tectopulvinar terminals, the synapsin content of RS, RM and RL profiles in the pulvinar nucleus and dorsal lateral geniculate nucleus (dLGN), and the properties of tectopulvinar EPSPs. We conclude that the short-term synaptic plasticity of RM profiles is distinct from that of RS and from that of RL profiles because each terminal type contains a unique distribution of synapsins. Furthermore, we suggest that the diffuse and specific tectopulvinar projections display subtle differences in short-term plasticity due to differences in glutamate release probability.

## Materials and Methods

A total of 16 tree shrews (*Tupaia belangeri*); 7 adults (more than 3 months old) and 9 juveniles (3 weeks old), were used for these experiments. Twelve tree shrews were used for in vitro physiology experiments, 1 tree shrew received injections of biotinylated dextran amine (BDA) in the SC, and tissue from 3 tree shrews was used for immunocytochemisty. In addition, we analyzed material from 2 tree shrews used in a previous study [9]. All procedures were approved by the Institutional Animal Care and Use Committee of the University of Louisville (Animal Welfare Assurance number A358601).

### Slice preparation

Thalamic slices (400 µm) were prepared from 9 juvenile and 3 adult male and female tree shrews using procedures previously described [22]. Briefly, the animals were deeply anesthetized with carbon dioxide and decapitated, the brain was excised and a block of tissue containing the thalamus was removed and placed in an ice-cold oxygenated solution of modified artificial cerebrospinal fluid (ACSF) containing (in mM) 206 sucrose, 2.5 KCl, 1 CaCl2, 1 MgSO4, 1 MgCl2, 1.25 NaH2PO4, 26 NaHCO3 and 10 glucose at a pH of 7.4 and equilibrated with 95% O2/5% CO2. Parasagittal slices were cut on a vibratome (Leica, VT 1000E, Deerfield, IL) at a thickness of 400 µm and transferred into a holding chamber with ACSF containing (in mM) 124 NaCl, 2.5 KCl, 2 CaCl2, 1 MgSO4, 1.25 NaH2PO4, 26 NaHCO3 and 10 glucose at a pH of 7.4 and equilibrated with 95% O2/5% CO2, where they incubated for at least 2 hours before recording.

### Electrophysiology

Whole cell recordings were obtained from the pulvinar nucleus. All recordings were carried out in the recording chamber maintained at 33°C with ACSF (with 10 µM bicuculline and 2.5 µM CGP55845 added; Tocris; Ellisville, MO) continually superfused at a rate of 2.0 ml/min. Pipettes were pulled from borosilicate glass (Sutter Instrument, Novato, CA) and had a tip resistance of 4–6 MΩ when filled with a solution containing (in mM) 115 K-gluconate, 2 MgCl2, 10 HEPES, 10 sodium phosphocreatine, 2 Na2-ATP, 20 KCl, 0.3 Na2-GTP with pH adjusted to 7.2 with KOH (osmolarity 290–295 mOsm). Biocytin (0.5%) was added to allow morphological reconstruction of the recorded neurons. Current-clamp recording were made with an Axoclamp 2B amplifier (Axon Instruments, Foster City, CA); the bridge was continually monitored and adjusted as needed. Data were digitized and stored on an IBM-compatible computer for off-line analyses. Only recordings showing a stable resting membrane potential more negative than -50 mV and over shooting action potentials were included in this study (n = 46). To stimulate the tectothalamic fibers a multipolar stimulation electrode (matrix microelectrode; FHC, Bowdoin, ME) was placed in the superficial layers of the superior colliculus (SC). Stimulating electrodes were at least 2 mm away from the recording electrode. Once a stable whole-cell recording was obtained, paired-pulse or train stimulation was produced by using any two adjacent electrodes (115 µm apart) in the arrays until the best response was achieved.

### Histochemistry

A subset of neurons was filled with 0.5% biocytin by diffusion from the pipette during recording (20 recovered). At the end of each recording, slices were fixed at 4°C overnight in 4% paraformaldehyde and rinsed several times in 0.1 M phosphate buffer (PB). Slices were then incubated in 10% methanol in PB with 3% hydrogen peroxide to react with the endogenous peroxidase activity of red blood cells. After several rinses in PB, slices were incubated overnight at 4°C under agitation in a 1% solution of avidin and biotinylated-horseradish peroxidase (ABC Kit Standard, Vector Laboratories) prepared in 0.3% Triton X-100. The slices were subsequently rinsed, reacted with nickel-intensified 3,3′-diaminobenzidine (DAB) for 5 min, and washed in PB. After rinses in phosphate buffer, slices were mounted onto slides and reconstructed with a Neurolucida system (Micro Bright Field Inc., USA). In some cases, biocytin-filled neurons were revealed by incubating slices in a 1∶100 dilution of streptavidin conjugated to Alexa Fluor 546 (Molecular Probes, Eugene, OR) and confocal images of the cell were obtained using an Olympus Fluoview laser scanning microscope (BX61W1).

### Tracer injections

As described in detail in our previous study [9], single tracer injections in the SC label dense topographic clusters of terminals (“specific” terminals); the most medial SC projects to the Pd, while the central and lateral regions of the SC project to the Pc. In contrast, the “diffuse” terminals are nontopographic; injections in any part of the SC label sparsely-distributed terminals in the Pd [37]. Therefore, when BDA is injected into the medial SC, tracer-labeled diffuse and specific terminals overlap within the Pd. However, when BDA is injected into the central or lateral SC, diffuse terminals are labeled in the Pd, while specific terminals are labeled in the Pc, allowing analysis of these segregated populations. Therefore we placed injections into the central and/or lateral regions of the SC to enable us to examine the isolated “diffuse” and “specific” projections in the Pd and Pc respectively.

Tree shrews that received BDA (3,000 MW; Molecular Probes, Eugene, OR) injections were initially anesthetized with intramuscular injections of ketamine (100 mg/kg) and xylazine (6.7 mg/kg). Additional supplements of ketamine and xylazine were administered approximately every 45 minutes to maintain deep anesthesia through completion of the tracer injections. Prior to injection, the tree shrews were placed in a stereotaxic apparatus and prepared for sterile surgery. A small area of the skull overlying the superior colliculus was removed and the dura reflected, a glass pipette containing either BDA (5% in saline, tip diameter 3 µm) was lowered vertically and the tracer was ejected iontophoretically (2 µA positive current for 15–30 minutes) into the central and/or lateral SC. After a 7-day survival period, the tree shrews were given an overdose of ketamine (600 mg/kg) and xylazine (130 mg/kg) and were perfused through the heart with Tyrode solution, followed by a fixative solution of 4% paraformaldehyde.

The BDA was revealed by incubating sections in a 1∶100 dilution of avidin and biotinylated horseradish peroxidase (ABC; Vector Laboratories, Burlingame, CA) in phosphate-buffered saline (0.01 M PB with 0.9% NaCl, pH 7.4; PBS) with 1% normal goat serum (NGS) overnight at 4°C. The sections were subsequently rinsed, reacted with nickel-intensified 3,3′-diaminobenzidine (DAB) for 5 minutes, and washed in PB. For confocal microscopy, the BDA was revealed by incubating sections in a 1∶100 dilution of streptavidin conjugated to Alexa Fluor 546 (Molecular Probes, Eugene, OR).

### Immunohistochemistry

Three adult tree shrews were given an overdose of sodium pentobarbital (tree shrew) were perfused through the heart with Tyrode solution, followed by a fixative solution of 4% paraformaldehyde in 0.1 M PB. The brain was removed from the skull, sectioned into 50- µm-thick slices using a vibratome (Leica VT100E, Leica Microsystems, Bannockburn, IL) and collected in a solution of 0.1 M PB. The sections were incubated at 4°C overnight with one of the following antibodies: guinea pig monoclonal anti-vGLUT1 or anti-vGLUT2 (1∶5000, Chemicon Temecula, CA; catalogue #'s AB5905 and AB2251), rabbit polyclonal anti-synapsin I (1∶1000, Millipore, Billerica, MA; catalogue #AB1543P) or rabbit monoclonal anti-synapsin II (1∶500, Abcam, Cambridge, MA; catalogue #AB76494). The following day the sections were rinsed in PB and incubated for 1 hour in an anti-guinea pig antibody conjugated to Alexafluor-488 (1∶100, Invitrogen/Molecular Probes, Carlsbad, CA; catalogue # A11073) or anti-rabbit antibody conjugated to Alexafluor-546 (1∶100, Invitrogen/Molecular Probes, Carlsbad, CA; catalogue # A11010). The sections were subsequently rinsed in PB and mounted on slides for confocal microscopic examination.

### Ultrastructural Analysis

Electron micrographs of diffuse and specific tectopulvinar terminals labeled by the anterograde transport of BDA injected into the SC, which were collected in a previous study [9], were re-analyzed. As described above, following BDA injections in the central and/or lateral SC, “diffuse” terminals were labeled in the Pd and “specific” terminals were labeled in the Pd. Resin-embedded sections were first examined with a light microscope to select “specific” terminals in the Pc and “diffuse” terminals in the Pd for electron microscopic analysis. Selected areas of the Pc or Pd were mounted on blocks, ultrathin sections (70–80 nm, silver-gray interference color) were cut using a diamond knife, and every fifth section was collected on Formvar-coated nickel slot grids. One in four of the collected sections was subsequently stained for the presence of gamma amino butyric acid (GABA) using gold particles, and examined using an electron microscope. Within each examined section, all labeled terminals involved in a synapse were photographed. The section spacing ensured that no single terminal was examined more than once. In the current study, we used ImageJ software (Rasband, W.S., U. S. National Institutes of Health, Bethesda, Maryland, USA, http://rsb.info.nih.gov/ij/, 1997-2009.) to measure the length of each synaptic contact, and the area of each presynaptic BDA-labeled terminal, within the previously collected images.

### Statistical Analysis

Student t-tests were used to test for statistical significance. Quantitative data are expressed as means ± SD. The significance level was set at p<0.05 for all statistical comparisons.

## Results

### Tectopulvinar clusters and convergence

**Figure 1:**
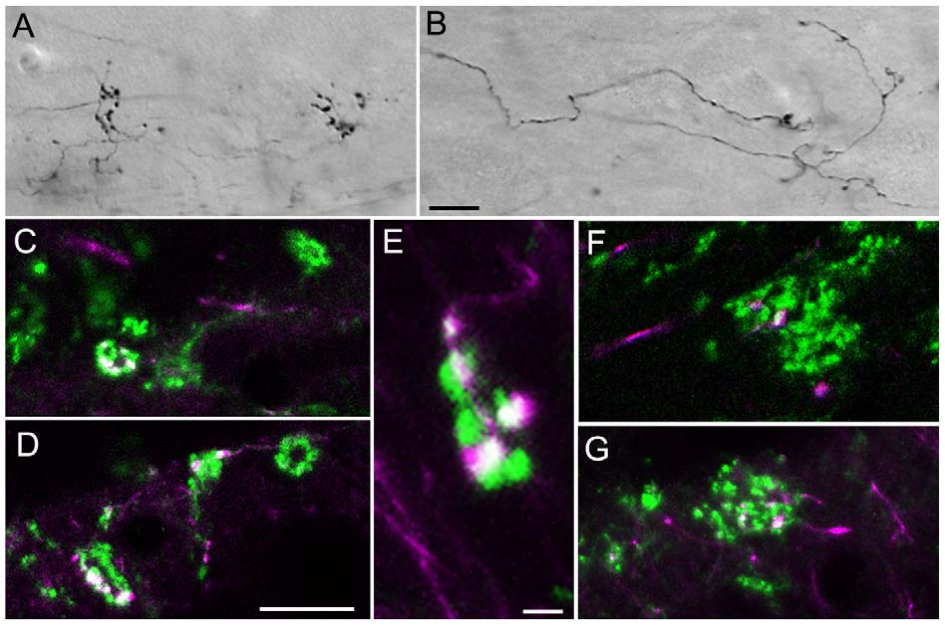
Convergence of tectopulvinar terminals. Injections of biotinylated dextran amine (BDA) in the SC labels tectopulvinar axons that form widespread (“diffuse”) axons and boutons as well as more discrete clustered (“specific”) boutons. Representative images of BDA-labeled “diffuse” (A) and “specific” (B) axons in 50 µm thick sections are illustrated using transmitted light, a 40x objective, and Nomarski optics. C–G) Confocal images (single 0.1 µm scan with a 100x objective) illustrate tectopulvinar terminals labeled with BDA (purple) and tectopulvinar terminals labeled with antibodies against the type 2 vesicular glutamate transporter (vGLUT2, green). Terminals labeled with both BDA and vGLUT2 appear white. Clusters of vGLUT2-stained terminals contain at most 1 bouton contributed by “diffuse” axons (F), while “specific” axons contributed several boutons to each cluster (C-E, G) Scale in B = 10 µm and applies to A. Scale in D = 10 µm and applies to C, F and G. Scale in E = 2 µm.

As previously reported [9], injections of biotinylated dextran amine (BDA) in the SC label tectopulvinar axons that terminate in nontopographically organized widespread boutons (“diffuse” projections; Figure 1A) as well as topographically organized clusters of boutons (“specific” projections; Figure 1B). By comparing BDA-labeled tectopulvinar synapses to synapses labeled by the vGLUT2 antibody (a marker for tectothalamic terminals; [9], [10]), we previously concluded that both the diffuse and specific projections are convergent [9]. We confirmed this by staining tissue that contained BDA-labeled tectopulvinar axons with the vGLUT2 antibody. As illustrated in Figure 1C–G, for both types of tectopulvinar axons, the BDA-labeled terminals made up a small proportion of the total terminals within a vGLUT2-stained cluster. However, vGLUT2-stained clusters contained at most 1 bouton contributed by “diffuse” axons (Figure 1F), whereas “specific” axons contributed several boutons to each cluster (Figure 1C–E, G). This suggests the “diffuse” pathway exhibits a greater degree of convergence onto individual dendrites than does the “specific” pathway.

### Specific synapses are longer than diffuse synapses

**Figure 2:**
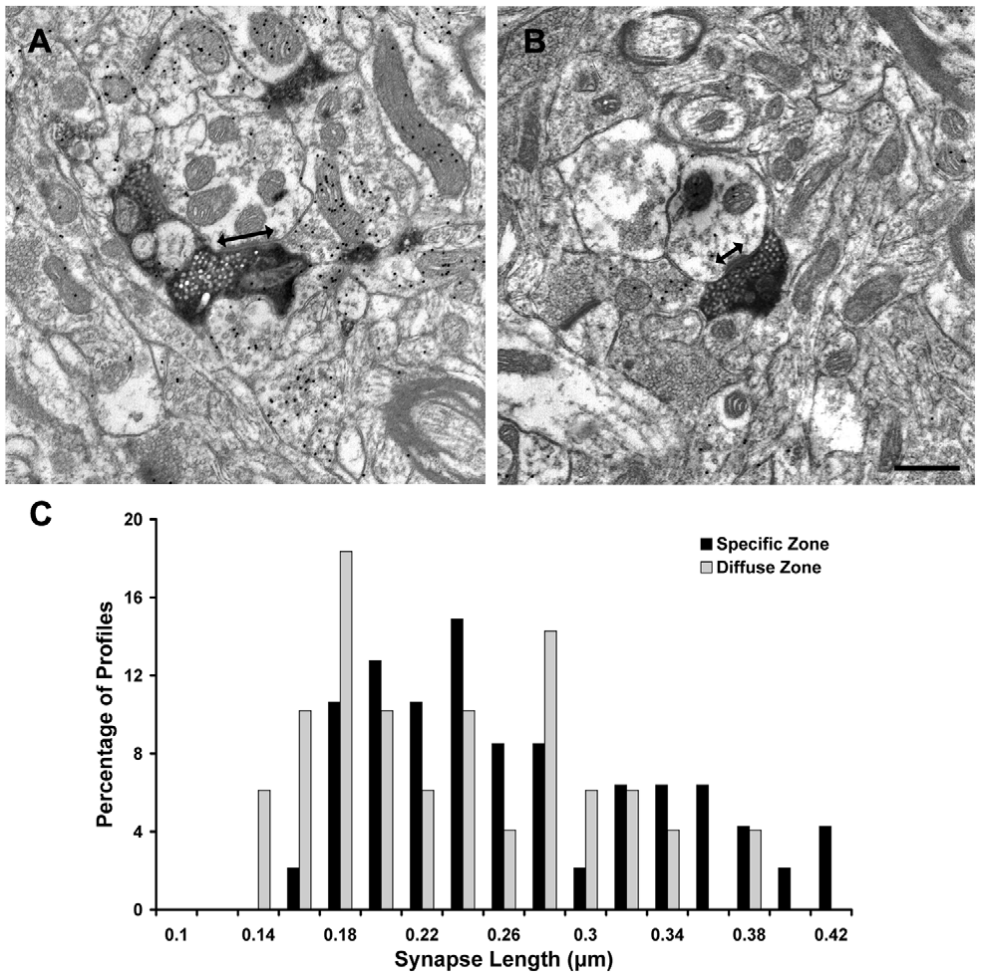
“Specific” tectopulvinar synapses are longer than “diffuse” tectopulvinar synapses. Electron micrographs illustrate examples of specific tectopulvinar terminals in the Pc (A) and diffuse tectopulvinar terminals in the Pd (B) labeled by the anterograde transport of biotinylated dextran amine (BDA) from the superior colliculus. The synapse length (arrows) of each terminal type was measured. The distribution of synapse lengths is plotted (C). As a population, the length of specific synapses was found to be significantly longer than the length of diffuse synapses (P<0.05). Scale bar  =  0.5 µm.

We re-analyzed electron micrographs collected in our previous ultrastructural study of tectopulvinar terminals labeled by the anterograde transport of BDA injections into the SC [9] to compare the synapse length of “specific” terminals (Figure 2A, n = 47) imaged within Pc tissue and “diffuse” terminals (Figure 2B, n = 49) imaged in Pd tissue. As illustrated in Figure 2C, the distribution of the synapse lengths overlapped, but as a population, specific synapses (0.25±0.07 µm) were found to be significantly longer than diffuse synapses (0.22±0.07 µm, P<0.05). We found no significant difference in the size of the specific and diffuse terminals measured at the site of synaptic contacts (specific 0.46±0.21 µm2; diffuse 0.47±0.21 µm2). In this single section analysis most terminals made single synaptic contacts (46 in Pc, 47 in Pd), but a few made more than one contact (1 in Pc, 2 in Pd).

### Tectopulvinar terminals contain both synapsin I and synapsin II

**Figure 3:**
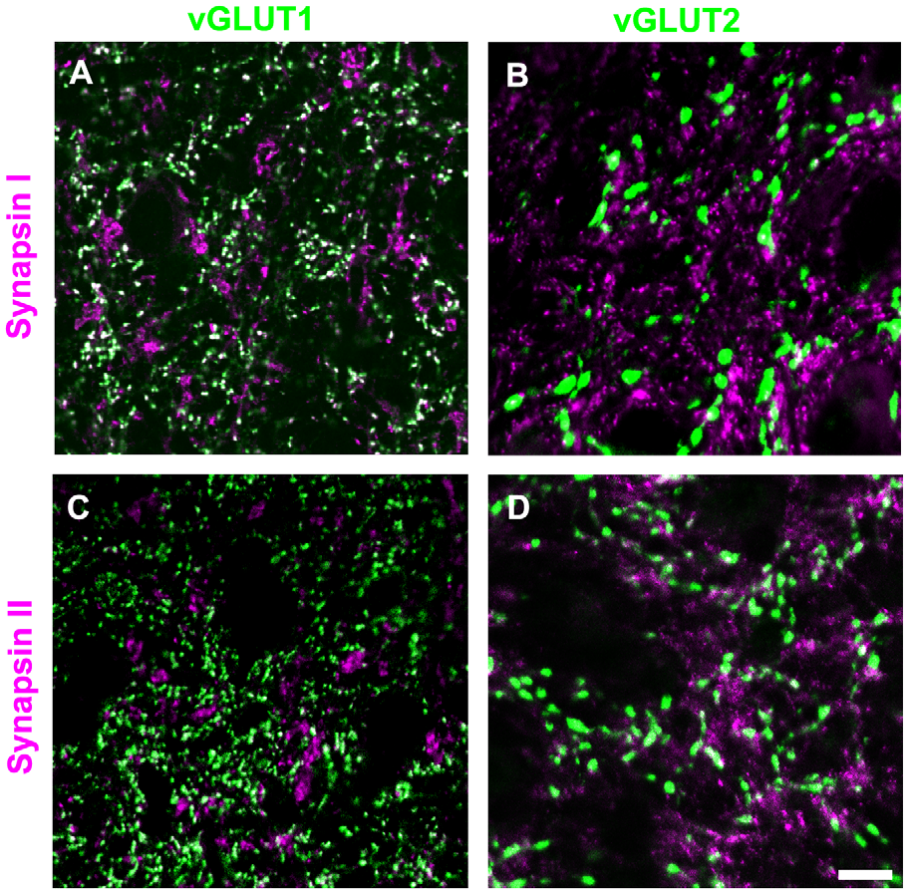
Distribution of synapsins and vGLUTs in the dLGN. Tree shrew dLGN tissue was labeled with antibodies against synapsin I or II (purple), and vGLUT1 or 2 (green). Profiles that are labeled with two antibodies appear white. Representative confocal images (single 0.2 µm scan with a 100x objective) are illustrated. Corticogeniculate terminals (RS profiles labeled with the vGLUT1 antibody) contain synapsin I (A), but not synapsin II (C). Retinogeniculate terminals (RL profiles labeled with the vGLUT2 antibody) do not contain either synapsin I (B) or synapsin II (D). Scale  =  10 µm.

**Figure 4:**
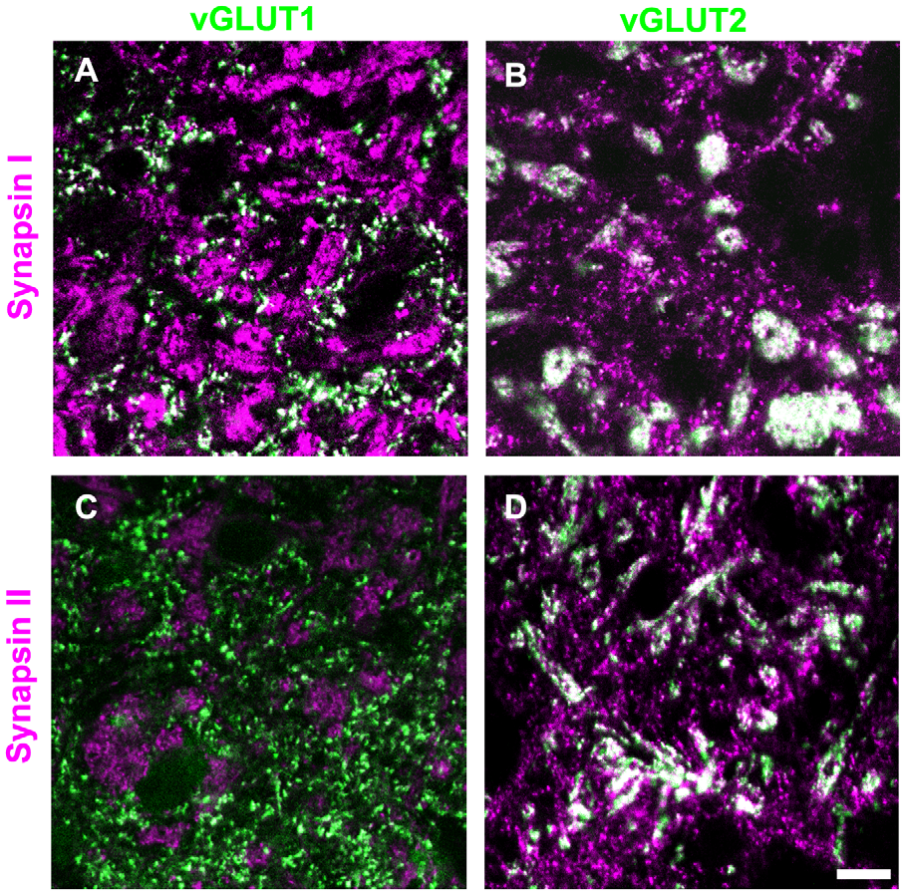
Distribution of synapsins and vGLUTs in the pulvinar nucleus. Tree shrew pulvinar tissue was labeled with antibodies against synapsin I or II (purple), and vGLUT1 or 2 (green). Profiles that are labeled with two antibodies appear white. Representative confocal images of the Pd (single 0.2 µm scan with a 100x objective) are illustrated. Corticopulvinar terminals (RS profiles labeled with the vGLUT1 antibody) contain synapsin I (A), but not synapsin II (C). Tectopulvinar terminals (large clusters of RM profiles labeled with the vGLUT2 antibody) contain both synapsin I (B) and synapsin II (D). Scale  =  10 µm.

Because the expression of synapsins has been related to short-term plasticity [36], we examined the synapsin content of RS, RM and RL profiles in the dorsal lateral geniculate nucleus (dLGN, Figure 3), and pulvinar nucleus (Figure 4) of the tree shrew by combining immunohistochemical labeling for synapsin I or synapsin II with immunohistochemical labeling for vGLUT2 (a marker for both tectothalamic and retinogeniculate terminals; [9], [10], [18]) or vGLUT1 (a marker for corticothalamic terminals; [39], [40], [41]). As illustrated in Figure 3, we found that retinogeniculate terminals (RL profiles labeled with the vGLUT2 antibody) do not contain either synapsin I or synapsin II (as was previously reported in the mouse; [36]). We also found that corticothalamic terminals in the dLGN and pulvinar nucleus (RS profiles labeled with the vGLUT1 antibody) contain synapsin I but not synapsin II (Figures 3–4). Finally, we observed that tectopulvinar terminals (RM profiles labeled with the vGLUT2 antibody) contained both synapsin I and synapsin II (Figure 4). We found a similar distribution of synapsins in vGLUT1- and vGLUT2-labeled terminals in the rat dLGN and lateral posterior nucleus (unpublished data).

Because tecopulvinar terminals form dense clusters, and individual terminals are difficult to distinguish at the light microscopic level, we used ImageJ software to quantify the labeling patterns by calculating the number of white pixels (double labeled areas) as a percentage of the total area labeled with the vGLUT antibodies (white + green pixels). For each image, we only analyzed areas that included both synapsin and vGLUT labeling to control for differences in antibody penetration. Three images of each antibody combination in the dLGN, Pd and Pc were analyzed.

This analysis confirmed our qualitative assessment. There was very little overlap of vGLUT2 in the dLGN (retinogeniculate terminals) with either synapsin I (1.62±0.40%) or synapsin II (1.99±0.43%). For vGLUT1 staining in the dLGN (corticogeniculate terminals) there was significant double-labeling with synapsin I (33.74±6.12%) but not with synapsin II (3.85±0.84%). This was also the case for vGLUT1 staining in the Pd and Pc (corticopulvinar terminals; Pd vGLUT1 29.36±1.54% overlap with synapsin I and 3.83±1.29% overlap with synapsin II; Pc vGLUT1 38.52±5.51% overlap with synapsin I and 3.24±1.29% overlap with synapsin II). In constrast, for vGLUT2 staining in the Pd and Pc (tectopulvinar terminals) there was significant double-labeling with both synapsins (Pd vGLUT2 57.86±2.45% overlap with synapsin I and 48.79±1.31% overlap with synapsin II; Pc vGLUT2 34.84±5.49% overlap with synapsin I and 47.83±4.25% overlap with synapsin II).

### In vitro recording of tectopulvinar synaptic potentials

In 400 µm thick parasagittal sections, whole cell recordings were obtained in the pulvinar nucleus and the rostral SC was stimulated to evoke EPSPs. The pulvinar EPSPs evoked by SC stimulation can be identified as tectopulvinar EPSPs because the cortical input to the dorsal (Pd) and central (Pc) pulvinar subdivisions originates exclusively from layer VI [6] while cortical input to the SC originates exclusively from layer V [42]. Therefore SC stimulation cannot activate corticopulvinar inputs. In addition, the pretectum does not project to the Pd or Pc [9], so current spread to this region does not activate any pulvinar inputs. Finally, cholinergic inputs from the parabigeminal nucleus that travel through the superficial SC and optic tract to the dLGN [43] do not innervate the pulvinar nucleus [9].

**Figure 5:**
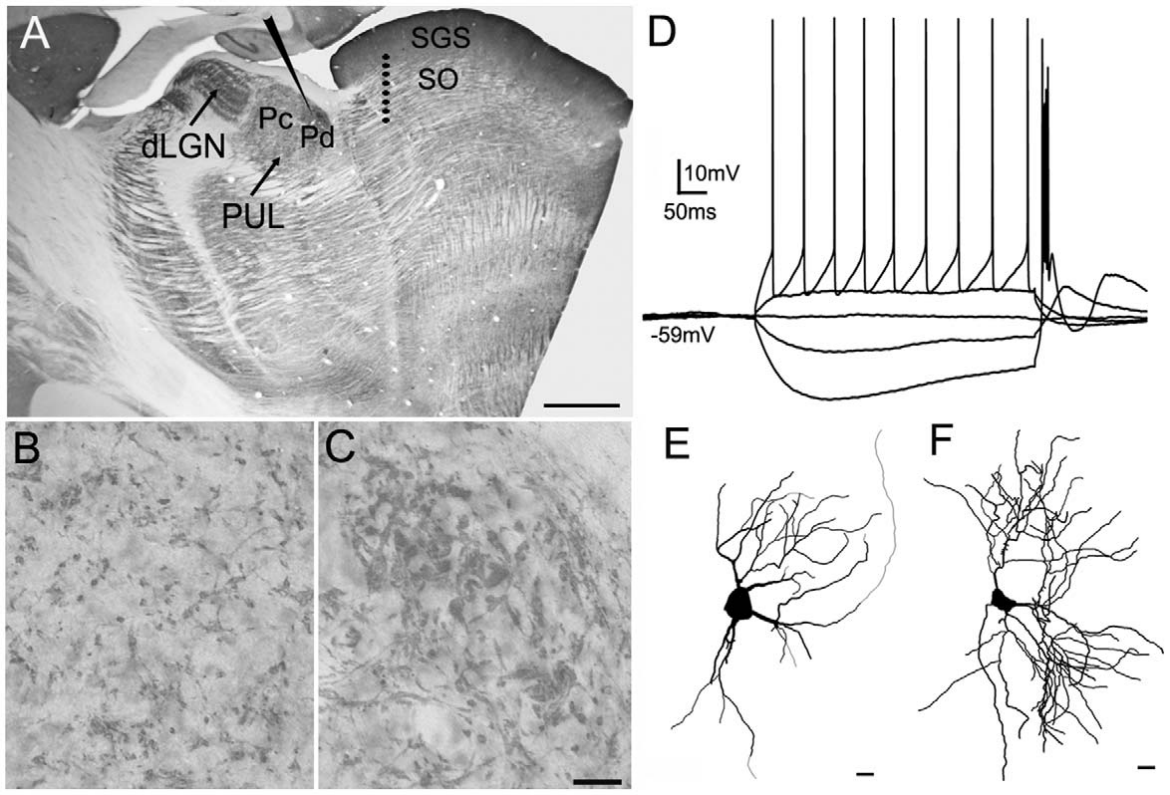
*In vitro* recording methods. A) A parasaggital section of the tree shrew brain stained for the type 2 vesicular glutamate transporter (vGLUT2) illustrates the location of the whole cell recordings in the caudal pulvinar nucleus and the location of the 8 electrode stimulus array in the stratum griseum superficial (SGS) and stratum opticum (SO) of the superior colliculus. B, C) High magnification views of the Pc (B) and Pd (C) illustrated in panel A. Immunohistochemical staining for vGLUT2 is a marker for tectopulvinar terminals [9] and reveals the distinct arrangements of tectal terminals in the dorsal (Pd) central (Pc) pulvinar nucleus. The Pd contains dense clusters of tectal terminals and tubular clusters line long lengths of dendrites (C). In contrast, the Pc contains smaller more sparsely distributed clusters of tectal terminals (B). D) Voltage fluctuations recorded in response to the injection of depolarizing or hyperpolarizing current steps of varying size revealed that all recorded cells fired with both tonic action potentials, and low threshold calcium bursts. E) Drawing of a biocytin-labeled cell recorded in the juvenile pulvinar, F) Drawing of a biocytin-labeled cell recorded in the adult pulvinar. Scale in A = 1 mm. Scale in C = 30 µm and also applies to B. Scales in E and F = 10 µm. dLGN, dorsal lateral geniculate nucleus.

Figure 5A illustrates the approximate location of the recording pipette in the pulvinar nucleus and the location of the 8 electrode stimulation array in the SC (spanning the superficial layers, stratum griseum superficiale and stratum opticum). The array spanned a distance of 1 mm and stimulation could be produced between any two electrodes in the array; the anode and cathode positions were varied to obtain the best response. With this configuration, tectopulvinar EPSPs could be elicited with a high rate of success (28 of 46 cells; 15 of 29 cells in juvenile tissue, and 10 of 17 cells in adult tissue) in the caudal pulvinar, which corresponds to the Pd (as described by Lyon et al., 2003[44]). In sections stained for vGLUT2, the Pd is identified as a region with densely distributed large clusters of terminals (Figure 5C) while the Pc contains smaller clusters of terminals that are more sparsely distributed (Figure 5D). We were unable to elicit tectopulvinar responses from more rostral regions of the pulvinar nucleus; in the parasagittal slice preparation, tectal axons traveling to the rostral pulvinar were most likely severed before they reached their synaptic targets.

As illustrated in Figure 5B, in response to the injection of depolarizing or hyperpolarizing current steps, all recorded neurons fired with either tonic action potentials or low threshold calcium spikes respectively, firing properties exhibited by thalamic relay cells [45]. Biocytin was included in the recording pipette and all recovered cells (n = 20) displayed morphologies consistent with their identification as relay cells (Figure 5E, F).

### Tectopulvinar EPSPs can be divided into two groups based on latency, and amplitude

**Figure 6:**
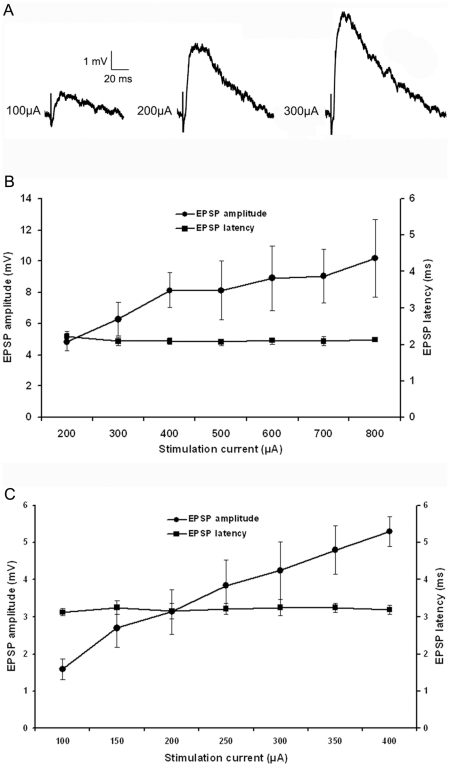
Two types of EPSPs. A) With increasing stimulation intensity, tecto-pulvinar EPSPs in the Pd show a graded increase in amplitude. B) Average first type EPSP amplitudes and latencies as a function of stimulation intensity (n = 17), graph show a graded increase in peak amplitude correlate to the increase in stimulation current but the latency of the EPSP is not relative to stimulation current. C) Average second type EPSP amplitudes and latencies as a function of stimulation intensity (n = 8), second type EPSP show a graded increase in peak amplitude and no change in latency with increasing stimulation intensity, but the threshold amplitude was smaller and latency was longer (p<0.05).

As illustrated in Figure 6, tectopulvinar EPSPs fell into two groups based on differences in their average latencies, as well as their threshold and peak amplitudes, suggesting that two different types of axon arbors were activated. EPSPs with the shortest latencies (2.11±0.10 ms, n = 17; 10 in juvenile tissue and 7 in adult tissue) exhibited the largest threshold EPSP amplitudes (4.82±0.57 mV, n = 17; Figure 6B) and EPSPs with longer latencies (3.20±0.15 ms, n = 8; 5 in juvenile tissue and 3 in adult tissue) exhibited smaller threshold amplitudes (1.58±0.27 mV, n = 8; Figure 6C). These parameters were found to be significantly different (latencies p<0.05; threshold amplitudes p<0.05), supporting the division of the tectopulvinar EPSPs into two groups. In addition, the rise time (10-90% of maximum amplitude) of short latency, large amplitude EPSPs (2.90±0.25 ms n = 17) was significantly faster than that of the slow latency, small amplitude EPSPs (3.82± 0.27 ms, n = 8, p<0.05).

### The two types of tectopulvinar EPSPs display distinct short term plasticity

**Figure 7:**
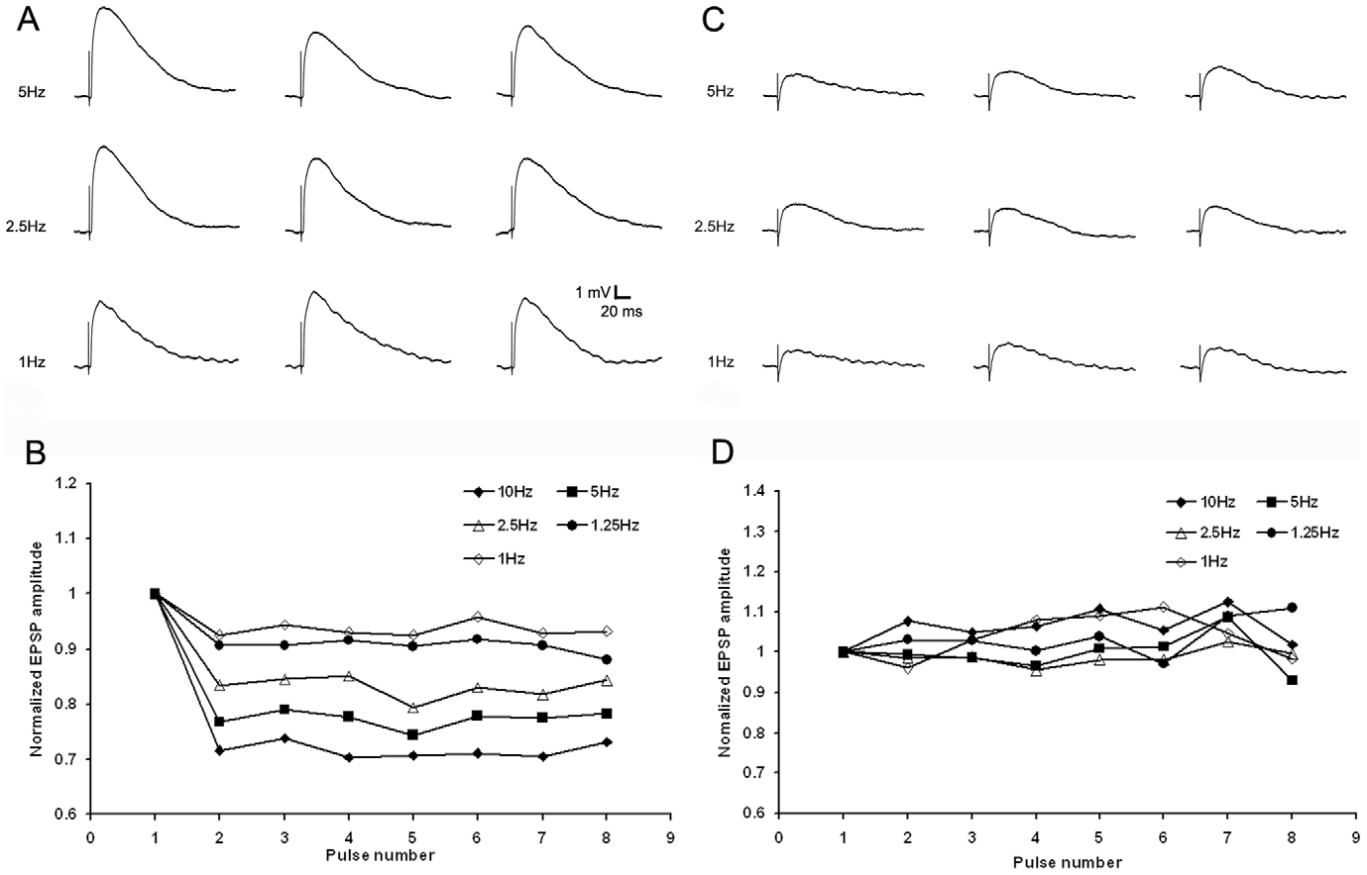
Two types of EPSPs display distinct frequency-dependent short-term plasticity. A) Train stimulation consisted of 8 pulses at 1 Hz, 1.25 Hz, 2.5 Hz, 5 Hz and 10 Hz, 1st, 3rd and 5th EPSPs from the train stimulation of first type tecto-pulvinar fiber showed frequency-dependent depression, recorded at resting membrane potential of -58mV. C) 1st, 3rd and 5th EPSPs from the train stimulation of second type tecto-pulvinar fiber produced EPSPs with stable amplitudes, recorded at resting membrane potential of −59 mV. B and D) Normalized average EPSPs evoked in 14 cells by 8 pulses at 1 Hz, 1.25 Hz, 2.5 Hz, 5 Hz and 10 Hz, each point was normalized by dividing the EPSP amplitude of that pulse in the train (EPSPn) to the amplitude of the first EPSP of the train (EPSP1), B) stimulation of the first type tecto-pulvinar fiber showed change in EPSP peak amplitudes of the 8 EPSPs evoked by the stimulus train at various frequencies. D) stimulation of the second type tecto-pulvinar fiber showed stable EPSP peak amplitudes of the 8 EPSPs evoked by the stimulus train at various frequencies.

**Figure 8:**
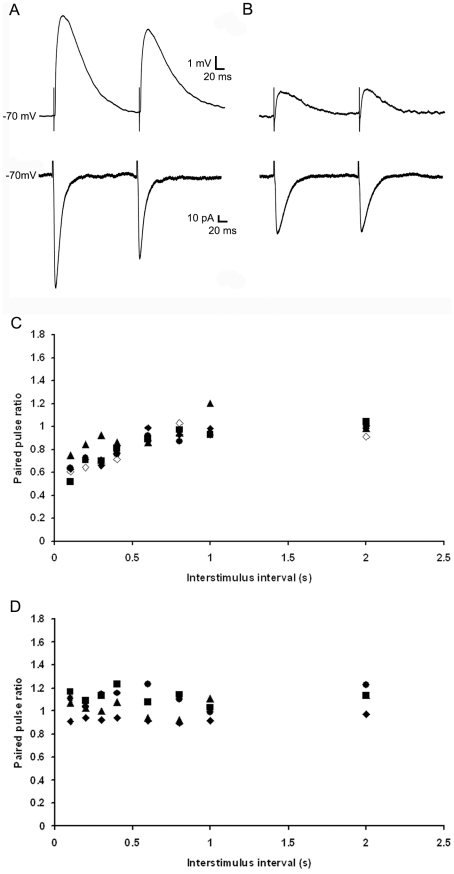
Two types of EPSPs display distinct short-term plasticity by paired-pulse stimulation. A) 1st and 2nd EPSP evoked by paired pulse stimulation of first type tecto-pulvinar fiber at 200 ms time interval, traces were recorded at resting membrane potential at −58 mV (upper panel) or −70 mV under voltage-clamp mode (lower panel), both traces showed paired-pulse depression. B) 1st and 2nd EPSP evoked by paired pulse stimulation of second type tecto-pulvinar fiber at 200ms time interval, traces were recorded at resting membrane potential at -59mV (upper panel) or −70 mV under voltage-clamp mode (lower panel), both traces showed stable EPSP amplitudes. C) Plots of paired-pulse ratio (EPSP2 amplitude/EPSP1 amplitude) as a function of inter-stimulus intervals (0.1 s, 0.2 s, 0.3 s, 0.4 s, 0.6 s, 0.8 s, 1 s, 2 s) by stimulation of first type tecto-pulvinar fiber, showed gradual increase of paired-pulse ratio. D) Plots of paired-pulse ratio (EPSP2 amplitude/EPSP1 amplitude) as a function of inter-stimulus intervals (0.1 s, 0.2 s, 0.3 s, 0.4 s, 0.6 s, 0.8 s, 1 s, 2 s) by stimulation of second type tecto-pulvinar fiber, showed stable paired-pulse ratio.

The short-term plasticity of these two types of EPSPs was tested with trains of stimuli varying in frequency from 1Hz to 10 Hz (Figure 7), as well as paired-pulse stimuli with interstimulus intervals of 0.1s to 2s (Figure 8). For each type of EPSP we used stimulation currents sufficient to evoke EPSPs at 50% of maximum. The short latency/large amplitude EPSPs were depressed by high frequency stimulation (frequencies >1 Hz, n = 10). With train stimuli, relative to the first EPSP of the train, the second EPSP decreased by 16.67±2.35% at 2.5 Hz, 23.29±3.88% at 5 Hz, and 29.42±3.42% at 10Hz (Figure 7A and B). Similar values were observed using paired-pulse stimuli (a 9.19±4.99% reduction in the amplitude of the second pulse relative to the first pulse at interstimulus intervals of 0.6s; 17.17±7.51% at 0.2s and 37.18±8.27% at 0.1 s; Fig. 8A and C, n = 5). In contrast, for the slower latency/smaller amplitude EPSPs, there was no correlation between mean amplitudes and stimulation frequencies or paired-pulse interstimulus intervals (Fig. 7C and D, n = 6; Fig. 8B and D, n = 4).

### The two types of tectopulvinar EPSP display different degrees of convergence

All recorded tectopulvinar EPSP amplitudes (n = 28) increased as the stimulation intensity was increased, suggesting that each postsynaptic cell receives input from multiple convergent tectal axons. However, the degree of convergence was found to be greatest for the slower/smaller/nondepressing EPSPs. The peak amplitudes of these EPSPs increased up to 3.34 fold above threshold amplitude values, while peak amplitudes of the faster/larger/depressing EPSPs increased to 2.11 fold above threshold values. As illustrated in Figure 6 (B and C), the rate of amplitude increase as a function of stimulation current was greater for nondepressing EPSPs compared to the depressing EPSPs.

### “Mixed” EPSPs

**Figure 9:**
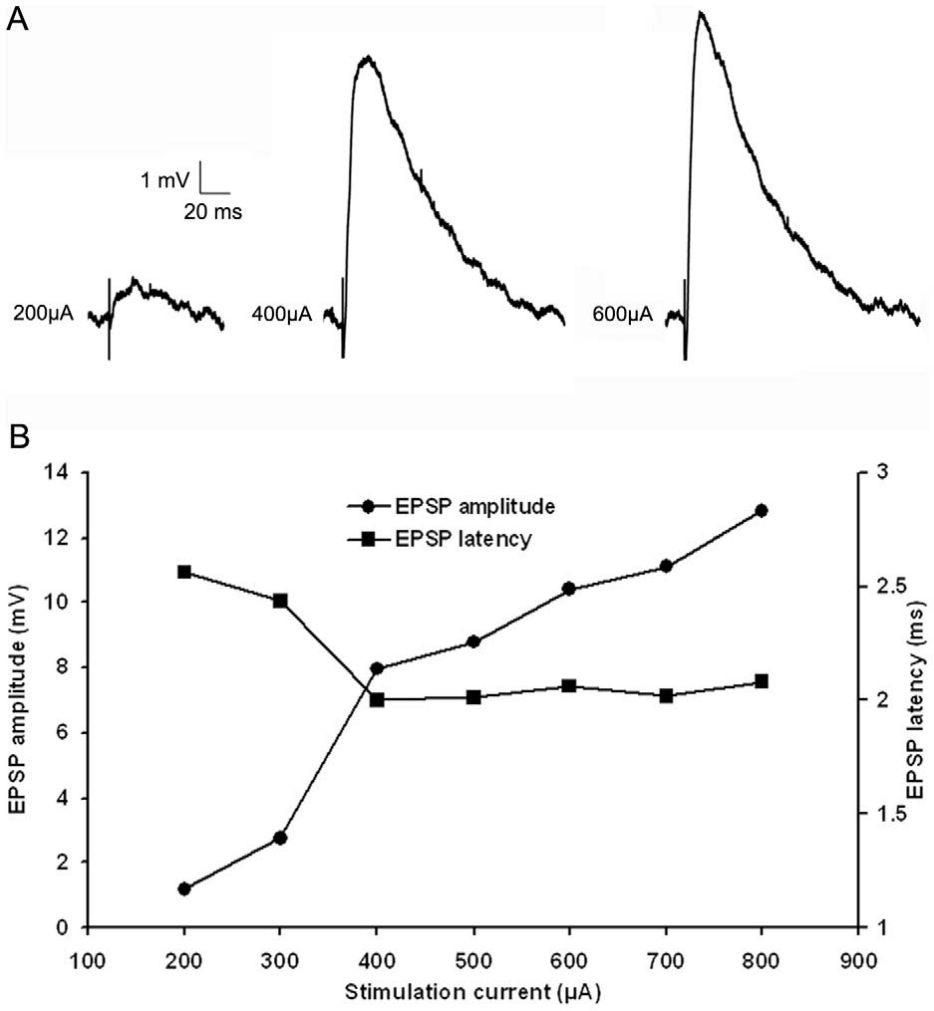
“Mixed” EPSP show characteristics of two types of EPSPs. A) “Mixed” EPSPs show small amplitude with small stimulation currents, followed by a large increase in amplitude and corresponding decrease in latency at larger stimulation currents. B) Plots of “mixed” EPSP amplitudes and latencies as a function of stimulation intensity, showed graded increase in amplitude with small stimulation currents followed by a large increase in amplitude and corresponding decrease in latency at larger stimulation currents.

For the majority of tectopulvinar EPSPs (25 of 28) the latencies did not change as the stimulation intensity was increased (Figure 6B, C). However, in some cases (n = 3) EPSPs appeared to contain a mixture of the two EPSP types described above. The amplitude of these “mixed” EPSPs initially increased gradually as the stimulation current was increased, but then exhibited a sudden large increase in amplitude, suggesting that the threshold was reached to recruit a different type of axon. Supporting this conclusion, there was a corresponding decrease in EPSP latency with the jump in EPSP amplitude (Fig. 9, A and B). Furthermore, for “mixed” EPSPs the amplitudes were unaffected by stimulus frequency when stimuli were delivered with threshold current levels, but frequency-dependent depression was observed when the stimuli were delivered with current levels that elicited the larger amplitude EPSPs.

## Discussion

### Diffuse and specific EPSPs

Based on differences in their convergence patterns, we suggest that the slower latency, smaller amplitude EPSPs represent activation of diffuse tectopulvinar projections, while the faster, larger amplitude EPSPs represent activation of specific tectopulvinar projections. This suggestion is also based on comparisons to our previous study of EPSPs elicited by stimulation of tectothalamic axons in the rat. Tectothalamic projections in the rodents are nontopographic [46] and EPSP amplitudes remain stable at stimulation frequencies of up to 20 Hz [22]. The lack of short-term frequency-dependent plasticity in the rat tectothalamic pathway compares to the slower latency, smaller amplitude tectopulvinar EPSPs of the tree shrew. In contrast, the faster, larger amplitude tectopulvinar EPSPs of the tree shrew exhibited a small frequency-dependent depression (29.42% at 10Hz), a feature which was not observed in our studies of the rat tectothalamic EPSPs. This difference does not appear to be a function of age because we detected these two types of tetctopulvinar EPSPs in both juvenile and adult tree shrew tissue.

At threshold, specific EPSPs may be larger than diffuse EPSPs because stimulation of a single specific axon activates clusters of terminals that innervate the same dendrite [9], while stimulation of a single diffuse axon activates unitary inputs. In addition, relative to diffuse terminals, the glutamate release is likely to be higher for specific terminals because their synaptic zones are significantly larger. This conclusion is tentative since we measured the length of specific synapses in the Pc, while we recorded EPSPs in the Pd. However, assuming specific synapses are similar in the Pc and Pd, their longer length relative to diffuse synapses would help to explain why we observed frequency-dependent depression in one group of EPSPs; the pool of synaptic vesicles ready for release in specific tectopulvinar terminals may not be fully replenished during short interstimulus intervals. Diffuse tectopulvinar EPSPs may exhibit relative stability due to a lower glutamate release probability. Since we detected no difference in the overall size of specific and diffuse terminals, a lower probability of neurotransmitter release would imply less vesicle depletion.

### Synapsin distribution varies in glutamatergic terminals of the thalamus

Synapsins have been associated with the tethering of synaptic vesicles to cytoskeletal elements [47], [48], [49], [50], [51], [52], [53], [54] and are thought to regulate the equilibrium of a reserve pool of synaptic vesicles and a population of vesicles that are docked for ready release [55], [56]. It has been hypothesized that synapsins are necessary to sustain the release of neurotransmitter at high rates of synaptic transmission [50], [51], [52]. We found that, within the visual thalamus, tectothalamic terminals are the only glutamatergic terminals that contain both synapsin I and synapsin II. Retinogeniculate terminals contained neither synapsin, and corticothalamic terminals contained synapsin I, but not synapsin II. These distributions provide further support for the division of thalamic glutamatergic terminals into 3 functional classes, and are presumably related to the distinct synaptic properties of these terminal types. Retinogeniculate EPSPs exhibit strong frequency-dependent depression [25], [26], [27], corticothalamic EPSPs exhibit strong frequency-dependent facilitation [25], [29], [30], while tectothalamic EPSP amplitudes remain relatively stable during high frequency stimulation [22]. Even the tectopulvinar EPSPs that showed a small decrease in amplitude with high stimulation frequencies (29.42% decrease at 10Hz) stimulation are more stable than retinogeniculate EPSPs which can depress by as much as 60% at 10Hz [25].

Recent studies have suggested that synapsin IIa is a key regulator of the reserve pool of synaptic vesicles. Gitler et al. [35] found that synapsin IIa was the only synapsin isoform that could increase the reserve pool of synaptic vesicles and slow synaptic depression in neurons obtained from synapsin I/II/III triple knock-out mice. The synapsin II content of tectopulvinar terminals may make them particularly resistant to fatigue and able to follow high rates of firing. Synapsin I in tectothalamic terminals may additionally regulate plasticity. In the dLGN of synapsin I/II knockout mice, the short-term frequency-dependent facilitation of corticogeniculate EPSPs was increased, while long-term post-tetanic potentiation was decreased [36]. This change presumably occurred because fewer vesicles were tethered to retain a reserve pool. If this is correct, we would predict that tectothalamic EPSPs in synapsin I/II knockout mice would have larger initial amplitudes compared to controls, but would depress with high frequency stimulation. However, direct comparison of corticothalamic and tectothalamic EPSP characteristics attributable to synapsin content is complicated by the fact that other proteins are likely involved in the frequency-dependent facilitation of corticothalamic EPSPs. For example, growth associated protein 43 is distributed specifically in RS profiles [57] and may mediate an activity-dependent enhancement of neurotransmitter release [58], [59].

### Functional implications

Tree shrew tectopulvinar neurons are located in the lower stratum griseum superficiale (SGS) of the SC and have been termed wide-field vertical cells due to their widespread, obliquely-oriented dendrites that extend throughout the retinorecipient SGS [9], [60]. Neurons in the lower SGS of the tree shrew have large receptive fields, exhibit brisk responses to movement and are direction selective [61]. Firing rates of up to 250 Hz have been recorded in the tree shrew SGS. Moreover, many cells exhibit sustained activity as the leading or trailing edge of a stimulus moves across their receptive field [61]. Therefore, tree shrew tectopulvinar terminals must be uniquely equipped to sustain transmission from the SC to pulvinar neurons. We suggest that the convergent synaptic arrangements, as well as the synapsin content of tectopulvinar terminals, allows these terminals to relay a dynamic range of visual signals from the SC to the pulvinar. These features may be particularly important for the reliable transfer of visual signals to initiate and guide the appropriate actions in response to the movements of predator or prey.

## References

[1] Guillery RW (1969). The organization of synaptic interconnections in the laminae of the dorsal lateral geniculate nucleus of the cat.. Z Zellforsch Mikrosk Anat.

[2] Wilson JR (1993). Circuitry of the dorsal lateral geniculate nucleus in the cat and monkey.. Acta Anat (Basel).

[3] Vidnyanszky Z, Hamori J (1994). Quantitative electron microscopic analysis of synaptic input from cortical areas 17 and 18 to the dorsal lateral geniculate nucleus in cats.. J Comp Neurol.

[4] Bourassa J, Deschenes M (1995). Corticothalamic projections from the primary visual cortex in rats: a single fiber study using biocytin as an anterograde tracer.. Neuroscience.

[5] Erisir A, Van Horn SC, Sherman SM (1997). Relative numbers of cortical and brainstem inputs to the lateral geniculate nucleus.. Proc Natl Acad Sci U S A.

[6] Chomsung RD, Wei H, Day-Brown JD, Petry HM, Bickford ME (2010). Synaptic organization of connections between the temporal cortex and pulvinar nucleus of the tree shrew.. Cereb Cortex.

[7] Robson JA, Hall WC (1977). The organization of the pulvinar in the grey squirrel (Sciurus carolinensis). II. Synaptic organization and comparisons with the dorsal lateral geniculate nucleus.. J Comp Neurol.

[8] Kelly LR, Li J, Carden WB, Bickford ME (2003). Ultrastructure and synaptic targets of tectothalamic terminals in the cat lateral posterior nucleus.. J Comp Neurol.

[9] Chomsung RD, Petry HM, Bickford ME (2008). Ultrastructural examination of diffuse and specific tectopulvinar projections in the tree shrew.. J Comp Neurol.

[10] Masterson SP, Li J, Bickford ME (2009). Synaptic organization of the tectorecipient zone of the rat lateral posterior nucleus.. J Comp Neurol.

[11] Ogren MP, Hendrickson AE (1979). The morphology and distribution of striate cortex terminals in the inferior and lateral subdivisions of the Macaca monkey pulvinar.. J Comp Neurol.

[12] Ogren MP, Hendrickson AE (1979). The structural organization of the inferior and lateral subdivisions of the Macaca monkey pulvinar.. J Comp Neurol.

[13] Brunso-Bechtold JK, Casagrande VA (1985). Ultrastructure of the developing tree shrew lateral geniculate nucleus.. Brain Res.

[14] Hamos JE, Van Horn SC, Raczkowski D, Sherman SM (1987). Synaptic circuits involving an individual retinogeniculate axon in the cat.. J Comp Neurol.

[15] Montero VM (1989). Ultrastructural identification of synaptic terminals from cortical axons and from collateral axons of geniculo-cortical relay cells in the perigeniculate nucleus of the cat.. Exp Brain Res.

[16] Li J, Wang S, Bickford ME (2003). Comparison of the ultrastructure of cortical and retinal terminals in the rat dorsal lateral geniculate and lateral posterior nuclei.. J Comp Neurol.

[17] Baldauf ZB, Chomsung RD, Carden WB, May PJ, Bickford ME (2005). Ultrastructural analysis of projections to the pulvinar nucleus of the cat. I: Middle suprasylvian gyrus (areas 5 and 7).. J Comp Neurol.

[18] Bickford ME, Wei H, Eisenback MA, Chomsung RD, Slusarczyk AS (2008). Synaptic organization of thalamocortical axon collaterals in the perigeniculate nucleus and dorsal lateral geniculate nucleus.. J Comp Neurol.

[19] Bickford ME, Slusarczyk A, Dilger EK, Krahe TE, Kucuk C (2010). Synaptic development of the mouse dorsal lateral geniculate nucleus.. J Comp Neurol.

[20] Sherman SM, Guillery RW (1998). On the actions that one nerve cell can have on another: distinguishing “drivers” from “modulators”.. Proc Natl Acad Sci U S A.

[21] Smith PH, Bartlett EL, Kowalkowski A (2007). Cortical and collicular inputs to cells in the rat paralaminar thalamic nuclei adjacent to the medial geniculate body.. J Neurophysiol.

[22] Masterson SP, Li J, Bickford ME (2010). Frequency-dependent release of substance P mediates heterosynaptic potentiation of glutamatergic synaptic responses in the rat visual thalamus.. J Neurophysiol.

[23] Granseth B, Ahlstrand E, Lindstrom S (2002). Paired pulse facilitation of corticogeniculate EPSCs in the dorsal lateral geniculate nucleus of the rat investigated in vitro.. J Physiol.

[24] Van Horn SC, Erisir A, Sherman SM (2000). Relative distribution of synapses in the A-laminae of the lateral geniculate nucleus of the cat.. J Comp Neurol.

[25] Turner JP, Salt TE (1998). Characterization of sensory and corticothalamic excitatory inputs to rat thalamocortical neurones in vitro.. J Physiol.

[26] Chen C, Blitz DM, Regehr WG (2002). Contributions of receptor desensitization and saturation to plasticity at the retinogeniculate synapse.. Neuron.

[27] Kielland A, Heggelund P (2002). AMPA and NMDA currents show different short-term depression in the dorsal lateral geniculate nucleus of the rat.. J Physiol.

[28] Li J, Guido W, Bickford ME (2003). Two distinct types of corticothalamic EPSPs and their contribution to short-term synaptic plasticity.. J Neurophysiol.

[29] Lindstrom S, Wrobel A (1990). Frequency dependent corticofugal excitation of principal cells in the cat's dorsal lateral geniculate nucleus.. Exp Brain Res.

[30] von Krosigk M, Monckton JE, Reiner PB, McCormick DA (1999). Dynamic properties of corticothalamic excitatory postsynaptic potentials and thalamic reticular inhibitory postsynaptic potentials in thalamocortical neurons of the guinea-pig dorsal lateral geniculate nucleus.. Neuroscience.

[31] Benfenati F, Valtorta F, Rubenstein JL, Gorelick FS, Greengard P (1992). Synaptic vesicle-associated Ca2+/calmodulin-dependent protein kinase II is a binding protein for synapsin I.. Nature.

[32] Rosahl TW, Geppert M, Spillane D, Herz J, Hammer RE (1993). Short-term synaptic plasticity is altered in mice lacking synapsin I.. Cell.

[33] Humeau Y, Doussau F, Vitiello F, Greengard P, Benfenati F (2001). Synapsin controls both reserve and releasable synaptic vesicle pools during neuronal activity and short-term plasticity in Aplysia.. J Neurosci.

[34] Gitler D, Takagishi Y, Feng J, Ren Y, Rodriguiz RM (2004). Different presynaptic roles of synapsins at excitatory and inhibitory synapses.. J Neurosci.

[35] Gitler D, Cheng Q, Greengard P, Augustine GJ (2008). Synapsin IIa controls the reserve pool of glutamatergic synaptic vesicles.. J Neurosci.

[36] Kielland A, Erisir A, Walaas SI, Heggelund P (2006). Synapsin utilization differs among functional classes of synapses on thalamocortical cells.. J Neurosci.

[37] Luppino G, Matelli M, Carey RG, Fitzpatrick D, Diamond IT (1988). New view of the organization of the pulvinar nucleus in Tupaia as revealed by tectopulvinar and pulvinar-cortical projections.. J Comp Neurol.

[38] Day-Brown JD, Wei H, Chomsung RD, Petry HM, Bickford ME (2010). Pulvinar projections to the striatum and amygdala..

[39] Fremeau RT, Troyer MD, Pahner I, Nygaard GO, Tran CH (2001). The expression of vesicular glutamate transporters defines two classes of excitatory synapse.. Neuron.

[40] Herzog E, Bellenchi GC, Gras C, Bernard V, Ravassard P (2001). The existence of a second vesicular glutamate transporter specifies subpopulations of glutamatergic neurons.. J Neurosci.

[41] Kaneko T, Fujiyama F, Hioki H (2002). Immunohistochemical localization of candidates for vesicular glutamate transporters in the rat brain.. J Comp Neurol.

[42] Casseday JH, Jones DR, Diamond IT (1979). Projections from cortex to tectum in the tree shrew, Tupaia glis.. J Comp Neurol.

[43] Fitzpatrick D, Conley M, Luppino G, Matelli M, Diamond IT (1988). Cholinergic projections from the midbrain reticular formation and the parabigeminal nucleus to the lateral geniculate nucleus in the tree shrew.. J Comp Neurol.

[44] Lyon DC, Jain N, Kaas JH (2003). The visual pulvinar in tree shrews I. Multiple subdivisions revealed through acetylcholinesterase and Cat-301 chemoarchitecture.. J Comp Neurol.

[45] McCormick DA, Huguenard JR (1992). A model of the electrophysiological properties of thalamocortical relay neurons.. J Neurophysiol.

[46] Mooney RD, Fish SE, Rhoades RW (1984). Anatomical and functional organization of pathway from superior colliculus to lateral posterior nucleus in hamster.. J Neurophysiol.

[47] Schiebler W, Jahn R, Doucet JP, Rothlein J, Greengard P (1986). Characterization of synapsin I binding to small synaptic vesicles.. J Biol Chem.

[48] De Camilli P, Benfenati F, Valtorta F, Greengard P (1990). The synapsins.. Annu Rev Cell Biol.

[49] Benfenati F, Valtorta F, Greengard P (1991). Computer modeling of synapsin I binding to synaptic vesicles and F-actin: implications for regulation of neurotransmitter release.. Proc Natl Acad Sci U S A.

[50] Greengard P, Valtorta F, Czernik AJ, Benfenati F (1993). Synaptic vesicle phosphoproteins and regulation of synaptic function.. Science.

[51] Pieribone VA, Shupliakov O, Brodin L, Hilfiker-Rothenfluh S, Czernik AJ (1995). Distinct pools of synaptic vesicles in neurotransmitter release.. Nature.

[52] Rosahl TW, Spillane D, Missler M, Herz J, Selig DK (1995). Essential functions of synapsins I and II in synaptic vesicle regulation.. Nature.

[53] Gitler D, Xu Y, Kao HT, Lin D, Lim S (2004). Molecular determinants of synapsin targeting to presynaptic terminals.. J Neurosci.

[54] Samigullin D, Bill CA, Coleman WL, Bykhovskaia M (2004). Regulation of transmitter release by synapsin II in mouse motor terminals.. J Physiol.

[55] Llinas R, McGuinness TL, Leonard CS, Sugimori M, Greengard P (1985). Intraterminal injection of synapsin I or calcium/calmodulin-dependent protein kinase II alters neurotransmitter release at the squid giant synapse.. Proc Natl Acad Sci U S A.

[56] Hilfiker S, Pieribone VA, Czernik AJ, Kao HT, Augustine GJ (1999). Synapsins as regulators of neurotransmitter release.. Philos Trans R Soc Lond B Biol Sci.

[57] Bickford ME (1999). Growth-associated protein 43 is located in type I corticothalamic terminals in the cat visual thalamus.. J Neurosci.

[58] De Graan PN, Schrama LH, Heemskerk FM, Dekker LV, Gispen WH (1990). The role of protein kinase C substrate B-50 (GAP-43) in neurotransmitter release and long-term potentiation.. Adv Exp Med Biol.

[59] Benowitz LI, Routtenberg A (1997). GAP-43: an intrinsic determinant of neuronal development and plasticity.. Trends Neurosci.

[60] Graham J, Casagrande VA (1980). A light microscopic and electron microscopic study of the superficial layers of the superior colliculus of the tree shrew (Tupaia glis).. J Comp Neurol.

[61] Albano JE, Humphrey AL, Norton TT (1978). Laminar organization of receptive-field properties in tree shrew superior colliculus.. J Neurophysiol.

